# CABIN1 peptide effectively targets MEF2D-fusion protein in B-cell precursor acute lymphoblastic leukemia

**DOI:** 10.1038/s41392-025-02397-3

**Published:** 2025-09-15

**Authors:** Subin Cha, Sangho Lee, Han-Teo Lee, Hyonchol Jang, Hong-Duk Youn

**Affiliations:** 1 Stochastic Stemness Research Center, Department of Biomedical Sciences, Ischemic/Hypoxic Disease Institute, Seoul National University College of Medicine, Seoul, 03080 Republic of Korea; 2 Reasearch Institute, National Cancer Center, Goyang, 10408 Republic of Korea

**Keywords:** Haematological cancer, Drug development

**Dear Editor**,

Acute Lymphoblastic Leukemia (ALL) is a potent malignancy, constituting 30% of all pediatric cancers. B-cell precursor ALL (BCP-ALL), which represents over 80% of ALL cases, is distinguished by chromosomal rearrangements that yield chimeric fusion proteins, which are unique from their normal counterparts. Among these, myocyte enhancer factor 2D (*MEF2D*)-fusion genes are found in both pediatric and adult populations and are associated with notably poor prognoses.^1^ MEF2D-fusion proteins typically preserve the MADS-box and MEF2 domain from MEF2D at the N-terminus, with the C-terminus derived from the fusion partner (Fig. 1a). Given that MEF2D-fusion proteins are vital transcription factors for controlling the core regulatory circuit in pre-BCR oncogenic stemness^2^ and calcineurin binding protein 1 (CABIN1) inhibits the transcriptional activity of MEF2D,^3^ we hypothesize that CABIN1 could act as a potential inhibitor of MEF2D fusion driven BCP-ALL.

**Fig. 1:**
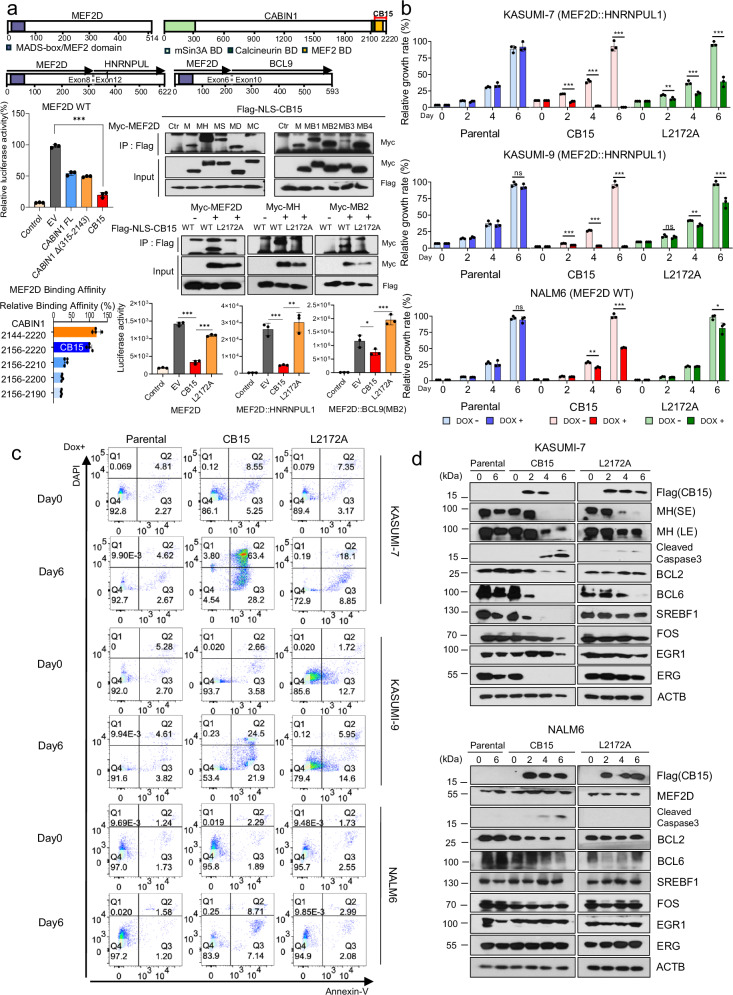
CB15 significantly impacts the survival of *MEF2D*-fusion BCP-ALL. **a** A schematic diagram illustrating the constructions of MEF2D-fusion forms and CABIN1 utilized in experiments. The fusion partners of MEF2D encompass HNRNPUL1 and BCL9. BD: Binding Domain (Middle, Left) Bar graphs represent luciferase activity as a proxy for transcription activity (means ± SD; *n* = 3). These data indicate that CB15 more effectively inhibits MEF2D-dependent transcription activity than either CABIN1 full-length or CABIN1 Δ315-2143. (Bottom, Left) The binding affinity between GAL4-MEF2D and VP16-CABIN1 peptides was observed using a mammalian two-hybrid system. pVP16-CABIN1 constructs with 10-amino acid truncations were transfected into HEK293T cells in conjunction with pGAL4-MEF2D under a Gal-promoter-luciferase reporter system (Promega) to evaluate binding affinity with luciferase activity. (means ± SD; *n* = 4). (Middle, Right, First) CB15 binds to all the MEF2D-fusion proteins. CB15 and MEF2D fusion proteins were co-transfected into HEK293T cells, and their interaction was verified by immunoprecipitation assays. The abbreviations are as follows: M, MEF2D; MH, MEF2D::HNRNPUL1; MS, MEF2D::SS18 (breakpoint involving exon 8 of MEF2D fused in frame to exon 6 of SS18); MD, MEF2D::DAZAP1 (breakpoint involving exon 6 of MEF2D fused in frame to exon 7 of DAZAP1); MC, MEF2D::CSF1R (breakpoint involving exon 7 of MEF2D fused in frame to exon 11 of CSF1R). MEF2D::BCL9 proteins are denoted as MB1 (breakpoint involving exon 6 of MEF2D fused in frame to exon 9 of BCL9), MB2, MB3 (breakpoint involving exon 5 of MEF2D fused in frame to exon 9 of BCL9), and MB4 (breakpoint involving exon 5 of MEF2D fused in frame to exon 10 of BCL9). (Middle, Right, Second) The interactions of CB15 with wild-type MEF2D and MEF2D fusion proteins were assessed via immunoprecipitation assays with HEK293T cells. The CB15 L2172A mutant exhibited reduced binding affinity to both wild-type MEF2D and MEF2D fusion proteins compared to the native CB15. (Bottom, Right) The direct binding of CB15 to MEF2D-fusion proteins influences their transcriptional activity. MEF2D-binding defective CB15 L2172A mutant exhibits weaker transcriptional inhibition than CB15 WT via a MEF2-luciferase assay. Bar graphs represent luciferase activity as a proxy for transcription activity (means ± SD; *n* = 3). Statistical comparisons were executed using two-tailed Student’s *t*-tests, with analyses conducted using GraphPad Prism 8. Significant differences were indicated as *p*-values: *p < 0.05, **p < 0.01, and ***p < 0.001. **b** The expression CB15 significantly reduced the growth rate in KAZUMI-7 cells. Cells were exposed to 200 ng/ml doxycycline for 6 days. Cell growth rate was assessed by measuring cell density every 2 days. (means ± SD; *n* = 3). Statistical differences were determined by the two-tailed Student’s *t*-test. *p* < 0.05 *, *p* < 0.01 **, *p* < 0.001 *** ns: not significant **c** The expression of CB15 significantly triggered cell death in KAZUMI-7 cells. Cells were exposed to doxycycline for 6 days. Cells were subjected to a flow cytometry analysis of apoptosis using Annexin-V conjugated Alexa-647 and DAPI dual staining. **d** Western blot analysis exhibits marker proteins indicating apoptosis and components of BCP-ALL core regulatory circuitry (CRC) during CB15 expression. ACTB served as a loading control. SE short exposure, LE long exposure

To confirm the optimal *CABIN1* sequence, we utilized a MEF2-luciferase reporter system in HEK293T cells. *MEF2D* transcriptional activity was evaluated by cotransfecting a MEF2 luciferase vector along with each of *CABIN1* Full Length (FL), *CABIN1* Δ315-2143, and *CABIN1*_15 (CB15, 2156-2220). *CABIN1* Δ315-2143, which contains binding domains with mSin3A and MEF2, was chosen based on the hypothesis that mSin3A’s interaction with HDAC might augment MEF2D transcriptional inhibition.^3^ *CB15*, a C-terminal fragment encompassing solely the core MEF2-binding sequence (2156-2190),^4^ excludes the calcineurin binding domain to potentially alleviate T cell-related side effects.^5^ Luciferase assays identified CB15 as the peptide effectively inhibiting the transcriptional activity of MEF2D (Fig. 1a). We sequentially truncated 10 amino acids from the C-terminus of *CB15* and measured MEF2D binding affinity of each truncated variant. Notably, the removal of just 10 amino acids significantly reduced the binding affinity (*p* < 0.001), indicating that these truncations render CB15 unsuitable for transcriptional repression (Fig. 1a). We verified CB15’s binding capacity to all observed MEF2D-fusion protein types^1^ in patients by immunoprecipitation assays (Fig. 1a). Cotransfection experiments with *CB15*, *MEF2D* WT, *MEF2D::BCL9*(MB2), and *MEF2D::HNRNPUL1*(MH) in HEK293T cells demonstrated that CB15 effectively suppresses transcriptional activity of all three types (Fig. 1a). The CB15 L2172A mutant, where the leucine residue crucial for MEF2 domain interaction^4^ is replaced with alanine, displayed decreased binding affinity to MEF2D-fusion forms and failed to inhibit transcriptional activity (Fig.1a). This confirms that CB15 inhibits the transcriptional activity of the MEF2D-fusion proteins through direct binding.

To examine the effects of CB15 on targeting the MEF2D-fusion protein, we constructed a doxycycline-inducible lentiviral system for expressing Flag-NLS-CB15/Flag-NLS-CB15 L2172A in *MEF2D::HNRNPUL1* BCP-ALL cells, KASUMI-7, and KASUMI-9 (Fig. 1b). The vectors designed to express TET3G and CB15 were engineered to constitutively express EGFP and mCherry, respectively, enabling us to confirm the correct construction of cell lines. To contrast the effects of CB15 on the MEF2D-fusion protein, we introduced the identical doxycycline-inducible system into the *MEF2D* WT BCP-ALL cell line NALM6 (Fig. 1b). We subsequently monitored alterations in cell growth over a 6-day period following doxycycline treatment. The growth rates of KASUMI-7 and KASUMI-9 cells were significantly reduced (*p* < 0.001) by CB15 expression compared to parental cells or cells expressing CB15 L2172A (Fig. 1b). Conversely, the growth rate of NALM6 cells was slightly impacted by CB15 expression and unaffected by CB15 L2172A expression (Fig. 1b).

To determine whether the inhibition of MEF2D-fusion proteins via CB15 triggers cell death, we conducted an apoptosis assay. By day6, over 90% of the CB15 expressing KASUMI-7 cells had undergone apoptosis whereas most parental cells remain viable (Fig. 1c). To a lesser degree, apoptosis was notably observed in CB15-expressing KASUMI-9 cells (Fig. 1c) whereas CB15 expression in NALM6 cells resulted in minimal apoptosis, affecting less than 16% of the cells (Fig. 1c). This stark contrast highlights the substantial impact of CB15 on the survival of *MEF2D*-fusion BCP-ALL cells. Additionally, CB15 L2172A is not as effective in inducing apoptosis as CB15 WT in both KASUMI-7 and KASUMI-9 (Fig. 1c). The inhibition of MEF2D-fusion protein expression by CB15 was also confirmed at the protein level. Consistent with apoptosis assays, CB15 expression in KASUMI-7 cells reduced MEF2D::HNRNPUL1 levels, increased Cleaved Caspase-3, and decreased BCL2. Furthermore, the expressions of BCL6, SREBF1, FOS, and EGR1, known as core regulatory circuit components critical for cell proliferation, differentiation blockade, and cell survival in BCP-ALL, also showed a decrease (Fig. 1d). ERG, a significant regulator of B-cell lineage genes, also exhibited a notable reduction in expression. In contrast, NALM6 cells exhibited no meaningful changes in these proteins upon CB15 expression (Fig. 1d). Moreover, the L2172A mutant in KASUMI-7 cells showed only minor differences in these proteins (Fig. 1d), suggesting that the direct binding of CB15 to the MADS-box/MEF2 domain could affect the survival of *MEF2D::HNRNPUL1* BCP-ALL cells. Our data propose CB15 as a potential novel agent for *MEF2D*-fusion BCP-ALL, a subtype currently lacking definitive treatment.^1^

We found that CB15 effectively targets the MEF2D fusion in *MEF2D::HNRN*PUL1 cell lines. Our results demonstrate that CB15 directly binds to the MEF2D fusion protein, thereby affecting cell survival and modulating the expression of core regulatory factors of BCP-ALL in the *MEF2D::HNRNPUL1* cell lines. The *MEF2D* fusion accounts for approximately 4% of childhood ALL cases, and patients with this fusion are classified as a high-risk group, experiencing a 5-year event-free survival rate of 71.6%.^1^ Notably, CB15 exerts its effects on cancer cells independently of T cell activation, suggesting its potential as a novel candidate with a different side effect profile compared to currently available agents. However, due to the limitations of in vitro cell line models, it is necessary to confirm whether CB15 can be applied to patient samples or preclinical models. Although CB15 clearly possesses inhibition potential, delivering the peptide into patient cells remains a major challenge. Future studies should explore feasible delivery strategies to realize CB15 as a therapeutic agent.

## Supplementary information

Supplementary Materials

## Data Availability

All data and materials supporting this study are available from the corresponding author upon reasonable request.
