# Supplementary material for: CABIN1 peptide effectively targets MEF2D-fusion protein in B-cell precursor acute lymphoblastic leukemia
Source: Signal Transduct Target Ther. 2025 Sep 15;10:294. doi: 10.1038/s41392-025-02397-3 (PMC12434140; doi:10.1038/s41392-025-02397-3)
Supplement: Supplementary file 1 — Supplementary Materials [file 41392_2025_2397_MOESM1_ESM.docx]

Supplementary Materials for

CABIN1 peptide effectively targets MEF2D-fusion protein in B-cell precursor acute lymphoblastic leukemia

Subin Cha,^1-3,*^ Sangho Lee,^1-3,*^ Han-Teo Lee,^1-3^ Hyonchol Jang,^4^ Hong-Duk Youn^1-3^

^1^Stochastic Stemness Research Center, ^2^Department of Biomedical Sciences, ^3^Ischemic/Hypoxic Disease Institute, Seoul National University College of Medicine, Seoul 03080, Republic of Korea; ^4^Reasearch Institute, National Cancer Center, Goyang 10408, Republic of Korea

*These authors are equally contributed.

Correspondence to: Hong-Duk Youn, Stochastic Stemness Research Center, Department of Biomedical Science, Seoul National University Medical Research Center, Seoul, 03080, Korea. Tel: +82-2-740-8250; Fax: +82-2-3668-7897; Email: [hdyoun@snu.ac.kr](mailto:hdyoun@snu.ac.kr)

**This PDF file includes:**

Materials and Methods

Materials and Methods

**Cell lines and reagents**

KASUMI-7, KASUMI-9 and NALM6 cells were maintained in RPMI 1640 (Cytiva, USA) supplemented with 10% or 20% fetal bovine serum (gibco, USA and Canada) at 37℃ in a humidified atmosphere containing 5% CO_2_. HEK293T cells were maintained in DMEM (Cytiva, USA) supplemented with 10% fetal bovine serum under the same conditions. KASUMI-7 and KASUMI-9 cells were purchased from Japanese Collection of Research Bioresources. NALM6 cells were obtained from ATCC. KASUMI-7 and KASUMI-9 cell lines were employed as pre-BCR-positive MEF2D BCP-ALL models^1,2^. In contrast, NALM-6 served as a pre-BCR-positive non-MEF2D BCP-ALL model, harboring a DUX4-IGH fusion rather than a MEF2D fusion^3^. Doxycycline (D981, Sigma), Ionomycin (I0634, Sigma).

**Antibodies**

anti-Flag (F3165, Sigma), anti-MEF2D (OAAB07955, Aviva), anti-Cleaved Caspase3 (9662S, Cell Signaling), anti-BCL2 (sc-7382, Santa Cruz), anti-BCL6 (#12895, Cell Signaling), anti-SREBF1 (14088-1-A, Proteintech), anti-FOS (ab7963, abcam), anti-EGR1(#4154, Cell Signaling), anti-ERG (sc-354(C-17), Santa Cruz), anti-Myc (626802(9E10), Biolegend), anti-ACTB (A5441, Sigma)

.

**Luciferase assay**

HEK293T cells were transiently transfected with expression vectors encoding luciferase, CABIN1 fragment, and MEF2D fusion protein. Cells were lysed with Potassium phosphate buffer supplemented with protease inhibitor cocktail and subjected to sonication (KORPROTECH, KFS-150N). Lysates were then centrifuged at 14,000 rpm for 10 minutes at 4℃. Luciferase activity was measured using a luminometer (Spark, Tecan) and analyzed with Magellan software.

**Flow cytometry analysis (Apoptosis assay)**

Cells(1x10^6) were incubated with Alexa Flour 647 conjugated Annexin V (A23204, Invitrogen), Annexin binding buffer and DAPI for 15 min on RT. Cells were subjected to flow cytometry using a flow cytometer (Becton Dickinson, BD LSRII (SORP)).

**Western blot**

After washing with PBS, cells were centrifuged at 5,000 rpm for 5 min. Subsequently, the cells were lysed using RIPA buffer supplemented with a protease inhibitor cocktail and transferred to 1.5 ml microtubes. Cells were sheared for 30 seconds per cycle, for a total of 5 cycles (Bioruptor Pico, Diagenode). Lysates were centrifuged at 14,000 rpm for 10 minutes at 4°C, and the cellular debris was discarded. For immunoprecipitation, cell lysates were incubated with Anti-FLAG M2 Affinity Gel (A2220, Sigma-Aldrich) at 4°C overnight. For immunoblotting, lysates were denatured in 1X sodium dodecyl sulfate (SDS) protein sample buffer at 100°C for 5 min and resolved by electrophoresis on 8% or 15% SDS-polyacrylamide gels. Separated proteins were transferred onto NC

Reference

1. Kasai, F. *et al.* Kasumi leukemia cell lines: characterization of tumor genomes with ethnic origin and scales of genomic alterations. *Hum. Cell* **33**, 868–876 (2020).

2. Leo, I. R. *et al.* Integrative multi-omics and drug response profiling of childhood acute lymphoblastic leukemia cell lines. *Nat. Commun.* **13**, 1691 (2022).

3. Tian, L. *et al.* Long-read sequencing unveils IGH-DUX4 translocation into the silenced IGH allele in B-cell acute lymphoblastic leukemia. *Nat. Commun.* **10**, 2789 (2019).
